# Management of scorpion envenoming: a systematic review and meta-analysis of controlled clinical trials

**DOI:** 10.1186/s13643-017-0469-8

**Published:** 2017-04-08

**Authors:** Chaturaka Rodrigo, Ariaranee Gnanathasan

**Affiliations:** Department of Clinical Medicine, Faculty of Medicine, University of Colombo, 25, Kynsey Road, Colombo, 08 Sri Lanka

**Keywords:** Scorpion, Prazosin, Antivenom, Controlled clinical trial, Systematic review

## Abstract

**Background:**

Scorpion stings cause an estimated 3000 deaths per annum worldwide. We conducted a systematic review of all controlled clinical trials related to scorpion sting management.

**Methods:**

We searched PubMed, EMBASE, Scopus, Web of Science and CINAHL and included controlled prospective clinical trials (randomized or non-randomized). The following interventions were assessed: adults and children with scorpion stings treated with (a) steroids vs. placebo, (b) different methods of pain relief, (c) antivenom vs. supportive treatment, (d) prazosin vs. supportive treatment, (e) antivenom vs. prazosin and (f) antivenom plus prazosin vs. prazosin alone. When trials had comparative outcomes, they were combined in a meta-analysis. Data was analysed with Review Manager 5. Dichotomous data were compared with relative risk (RR), and continuous data were compared with mean differences using a fixed effect model. There is no PROSPERO registration number for this study.

**Results:**

Antivenom against *Centruroides* sp. are effective in reversing the clinical syndrome faster than no antivenom treatment in children (RR, 0.02; 95% CI, 0.01 to 0.06; 322 participants; three trials). Antivenom (against *Mesobuthus tamulus)* and prazosin combination is better than prazosin alone for faster resolution of symptoms (mean difference, −12.59 h; 95% CI, −14.01 to −11.17; 173 participants; three trials).

**Conclusions:**

The polyvalent antivenom against *Centruroides* sp. in USA/Mexico and the monovalent antivenom against *M. tamulus* in India are effective for rapid resolution of symptoms. Prazosin is useful as an add-on therapy for *M. tamulus* stings.

**Electronic supplementary material:**

The online version of this article (doi:10.1186/s13643-017-0469-8) contains supplementary material, which is available to authorized users.

## Background

Scorpion stings are a cause of significant morbidity and mortality in Central and South America, Middle East, Africa and South Asia [1]. Interestingly, despite the wide geographical distribution of venomous scorpions, almost all species that are harmful to humans belong to the *Buthidae* family [1]. The scorpion has a stinger in their terminal segment that has venom glands and uses it to penetrate the skin of a potential target to inject venom. The syndrome of scorpion envenoming is less heterogeneous than snake envenoming with all the major manifestations being autonomic neuroexcitatory (stimulation of both sympathetic and parasympathetic systems). The main target of venom is voltage-gated sodium channels [2]. Once the scorpion venom peptides (scorpion α toxins) bind to these channels, their inactivation is inhibited leading to prolonged depolarization with neuroexcitation. Envenoming is characterized by autonomic disturbances such as tachy/bradycardia, hyper/hypotension, excessive salivation and lacrimation, urinary and faecal incontinence and pulmonary oedema. Deaths from scorpion stings are usually due to cardiogenic shock and pulmonary oedema [3]. Stings are rarely fatal in developed countries, but they are still a significant cause of mortality in developing nations. It is estimated that over one million scorpion stings (with approximately 3000 deaths) occur every year in endemic areas [4].

In this background it is essential to be clear about the usefulness of current treatment strategies for scorpion envenoming. The main modes of therapy for scorpion stings historically were supportive treatment and immunotherapy in form of antivenom. However, whether antivenom added any advantage to standard supportive therapy has been debated. The only meta-analysis published on the topic in 2011 concluded that there was no evidence for use of scorpion antivenom for old world scorpion stings, but appropriate antivenom therapy enhanced recovery in new world scorpion stings [5]. These observations were based on nine studies (four randomized controlled trials and five observational studies) enrolling 687 patients. Four years have elapsed since the publication of this meta-analysis, and immunotherapy is a rapidly advancing field with novel evidence that has to be taken in to account when recommending current treatment strategies. Some reviewers have also expressed concern over the generalizations made in this review as the scorpion species are heterogeneous, and therefore, the ‘presumed efficacy’ of antivenom cannot be generalized [6]. Also, it may not be prudent to combine studies that tested different antivenoms (raised against venom of different species in different geographical locations) for a meta-analysis.

In addition to re-addressing the research question of whether immunotherapy carries an advantage in scorpion stings with more strict criteria, we also look into other evidence-based treatment strategies that had been tested in controlled clinical trials to date on scorpion stings.

### Objectives

The objective of this study is to assess the usefulness of treatment strategies and supportive measures for symptom resolution in scorpion envenoming in children and adults using evidence from controlled clinical trials.

## Methods

### Eligibility criteria

We included controlled clinical trials (randomized or non-randomized) in this analysis. Case series where control populations were not available were excluded as they did not provide data on comparative efficacy. However, given the ethical constraints, we understand that it is difficult to enrol patients in to placebo arms for prospective trials. Therefore, we also included studies that compared prospectively enrolled patients with historical cohorts as controls. Level of evidence for recommendations was adjusted accordingly. Following a previously published review by Abroug et al., we analysed the trials for old world and new world scorpion stings separately [5].

### Information sources and search strategy

We searched PubMed, EMBASE, Scopus, Web of Science and CINAHL for relevant articles. PubMed was searched with the keywords ‘scorpion’ in abstract and ‘trial’ in any field without any language, time or other restrictions. EMBASE was searched with the keywords scorpion and trial in the title or abstract with no language or time limits. Scopus was searched with scorpion in title, keyword and abstract plus trial in title, keyword and abstract without any language or time limits. Web of Science and CINAHL were searched with scorpion in abstract and trial in any field. We used the software Endnote X3 (Thomson Reuters, Carlsbad, CA 92011, USA) to filter articles. The searches had a low specificity to not to miss any relevant articles. The search was repeated for all databases with keywords scorpion in title and abstract and ‘treatment’ or ‘treatment strategy’ in any field to find any missed articles with the previous strategy. Date of last search was 1st of December 2015. There is no PROSPERO registration number for this study. The PRISMA checklist for this review is given as an additional file (see Additional file 1: PRISMA checklist 2009.docx).

### Study selection and data collection

We read all abstracts independently and identified key articles by consensus. Depending on the abstracts, the papers were classified as ‘yes’ (meets inclusion criteria), ‘no’ (does not meet inclusion criteria) and ‘doubtful’. Full articles were obtained for all studies meeting the inclusion (or doubtful) criteria, and there were no articles in the doubtful category after reviewing the full articles. All identified controlled trials on scorpion stings were categorized according to themes of their research questions.

### Data items

The data items extracted from each eligible studies included participant demographics, intervention and control groups, drug doses, locality of study, species of scorpions studied, severity scoring of symptoms, duration for symptom resolution or improvement at 4 h since initiation of treatment, adverse events attributable to therapy and mortality rates.

### Risk of bias

We assessed the risks of bias of included studies qualitatively using the Cochrane risk of bias assessment tool [7]. We did not calculate quality scores for individual studies as it is not perceived by all as an objective measure of risk of bias [8].

### Summary measures and synthesis of results

When comparative trials were available to combine in a meta-analysis, we analysed the data using Review Manager 5 [7]. Dichotomous data were compared with relative risk (RR) and 95% confidence intervals (CI) and continuous data with mean differences. A fixed effect model was used for analysis. We assessed heterogeneity using the *I* ^2^ statistic [9]. This examines the percentage of total variation across studies that are due to heterogeneity rather than chance. When heterogeneity was present, a random effect model was used for analysis. The comparabilities and limitations of each study included in the meta-analyses is given in Table 1.

**Table 1:** Compatibilities and limitations for including studies in the meta-analysis

Clinical comparison and trials	Compatibilities	Limitations
Antivenom vs. placebo (old world scorpions)		
Abroug et al. [15] and Belghith et al. [16]	Both trials are from the same geographical region	Other treatment had been prescribed at clinicians discretion
	Both trials conducted in the same time frame	Findings are only applicable to the mentioned scorpion species and the particular antivenom tested
	The antivenom was produced against the same scorpion species: *A. australis* and *B. occitanus*	Both trials had been published over 15 years ago
	Dosage of antivenom was similar and was capable of neutralizing more than 10LD_50_ of venom/ml	
	Both trials were randomized prospective clinical trials	
	Severity grading of scorpion stings was similar	
Antivenom vs. placebo (new world scorpions)		
Two trials by Boyer et al. [19, 20] and one trial by LoVecchio et al. [18]	All three trials conducted in close geographical range (Arizona, USA and Mexico)	Severity grading on admission done by LoVecchio et al. only. Both other studies report venom concentration on admission by assays and give a descriptive analysis of symptoms
	All three trials used antivenom against *Centruroides* sp.	The neutralizing capacity of (LD_50_ equivalent in mice) administered antivenom reported in one trial only (Boyer et al. 2013)
	Symptom resolution at 4 h is reported in two trials and can be inferred by reported data in the third trial (LoVeccio et al.)	The differences in study design of the included studies are explained in Table 1
	All trials were on children or young adults (2 trials enrolled within an age range of 6 months to 18 years while the third included children under 2 years)	Trial findings and recommendations are limited to *Centruroides* sp. In Central America and Southern States of USA
Antivenom and prazosin vs. prazosin alone		
Bawaskar et al. [23], Natu et al. [22] and Pandi et al. [24]	All trials have been conducted in India	Heterogeneity in age groups (however, antivenom dosage was not dependent on age)
	All trials included antivenom against *M. tamulus*	Other differences in study design are explained in Table 2
	All trials report the mean time to resolution of symptoms	Two trials used the same protocol for antivenom administration and severity grading while the third (Natu et al.) used a different protocol
	All trials have used the same monovalent antivenom from the same company	Interpretations and recommendations limited to *M. tumulus* in India
	The maximum amount of venom injected per sting by *M. tamulus* is 1.5 mg and each ml of antivenom neutralized 1.2–1.5 mg of venom. All trials used at least 30 ml of venom per patient irrespective of the age	

### Risk of bias across studies

We could not construct funnel plots to assess publication bias as there were only a few studies per each comparison (less than three per comparison).

## Results

### Study selection

**Fig. 1:**
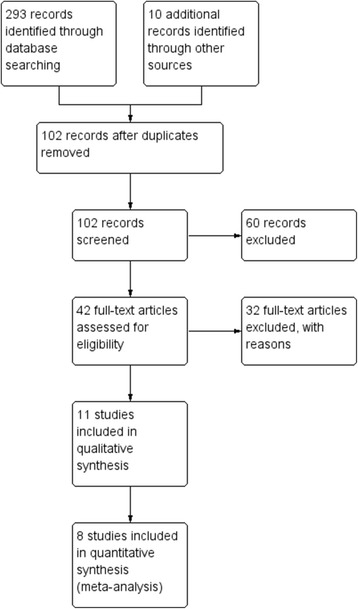
Flow diagram for selection of studies for the systematic review

A total of 293 studies were identified from database searches. After removal of duplicates and filtering according to eligibility criteria, 11 studies remained. Eight of these were included in the quantitative analyses (meta-analyses). A flow diagram gives the details of selection process (Fig. 1).

**Fig. 2:**
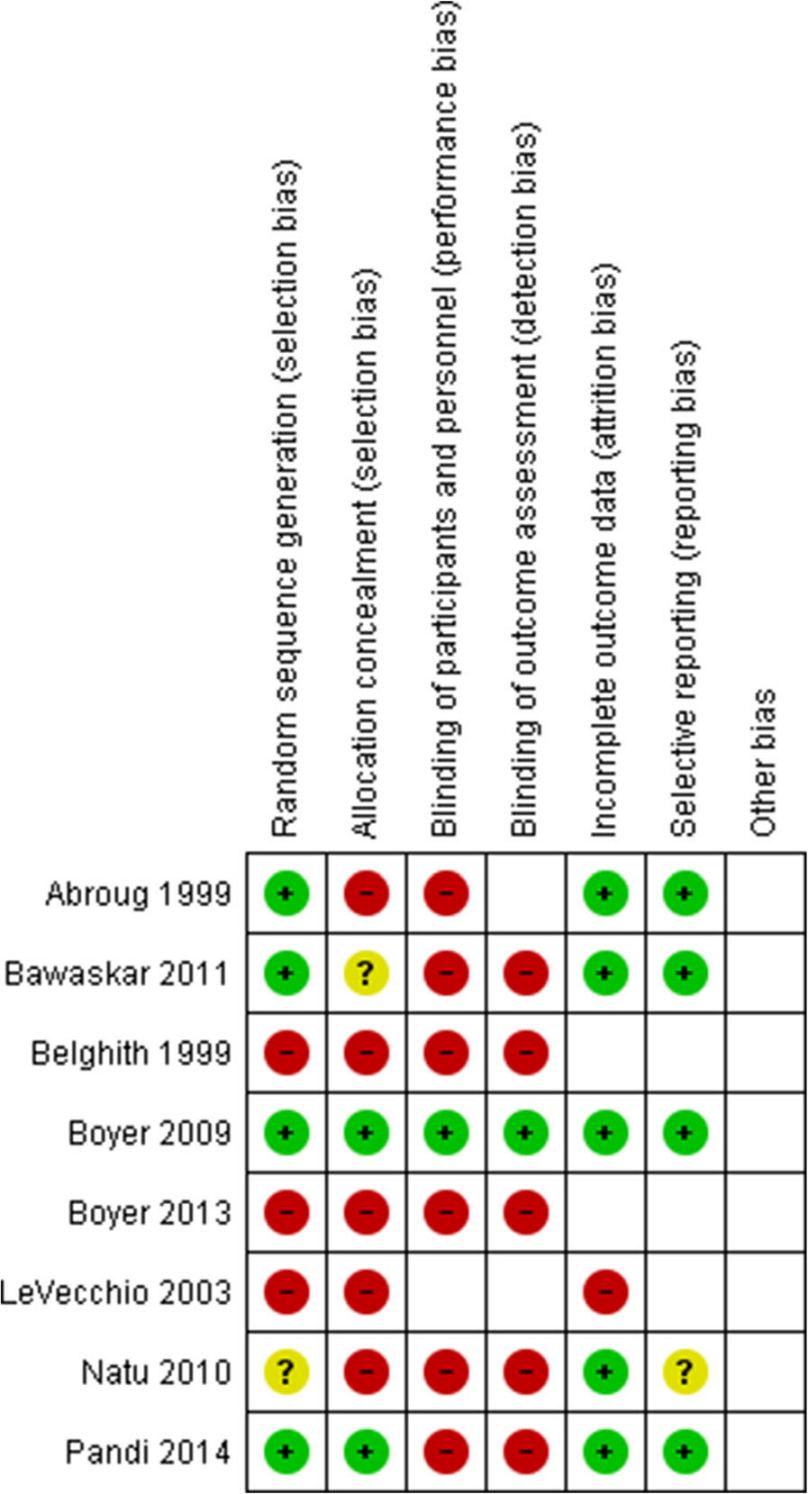
Risk of bias table for included studies (for meta-analyses)

The characteristics of included studies (for meta-analysis) are summarized in Table 2, and risk of bias of each study is summarized in Fig. 2.

**Table 2:** Characteristics of included studies for meta-analyses

Studies and parameters	Comments
Comparison	Antivenom vs. standard therapy (new world scorpions)
Boyer et al. 2009 [20]	
Methods	Randomized double blind study
Participants	Children and adults aged between 6 months and 18 years admitted to a paediatric intensive care unit within 5 h since a scorpion sting
Interventions	The test group (*n* = 8) received scorpion antivenom against *Centruroides* sp. while the control group (*n* = 7) received a placebo. Dose: three vials diluted in 50 ml of saline. Both groups received similar supportive care.
Outcomes	Resolution of clinical syndrome within 4 h of antivenom administration, cumulative midazolam dose required for sedation and serum venom levels up to 4 h post admission, adverse events
Study location	Arizona, USA
Boyer et al. 2013 [19]	
Methods	Controlled study with trial arm recruited prospectively and compared with a retrospective control group
Participants	Children and adults aged between 6 months and 18 years admitted to a paediatric intensive care unit within 5 h since a scorpion sting
Interventions	The prospectively recruited subjects (*n* = 78) received scorpion antivenom against *Centruroides* sp. while the control group (*n* = 97) had been managed without antivenom. Three vials of antivenom were administered within 10 min diluted in 50 ml of saline. Two additional vials were administered at the discretion of the physician if symptoms had not resolved at 1 or 2 h since the initial dose.
Outcomes	Resolution of clinical syndrome within 4 h of antivenom administration, serum venom levels up to 4 h post admission (in prospective group only), adverse events
Study location	Arizona, USA and Morelos, Mexico
LoVecchio et al. 2003 [18]	
Methods	Observational study without randomization
Participants	Children aged less than 2 years with either a witnessed scorpion sting or signs and symptoms consistent with scorpion envenoming
Interventions	Severity was graded on a scale of I–IV with grades III and IV having systemic envenoming. A subset of patients with grade III and IV envenoming had received anti-*Centruroides* antivenom (1 vial diluted in 50 ml of saline, *n* = 86). Another 46 children did not receive antivenom despite having grade III and IV envenoming. Criteria for administration of antivenom are not clear.
Outcomes	Meantime for resolution/improvement of systemic envenoming, adverse events, deaths
Study location	Arizona, USA
Comparison	Antivenom vs. standard therapy (old world scorpions)
Abroug et al. 1999 [15]	
Methods	Prospective randomized controlled trial
Participants	Patients with scorpion stings older than 10 years. A total of 825 patients were randomly allocated to test (*n* = 412) and control arms (*n* = 413)
Interventions	The test group received 20 ml of bivalent (*A. australis* and *B. occitanus*) scorpion antivenom. Both groups received supportive care with steroids, fluid replacement, antihistamines and life-supporting measures for systemic envenoming as required.
Outcomes	Severity was graded as I (no systemic envenoming) and II (systemic envenoming). Prevention was defined as non-progression from grade I to II, and cure was defined as reversal of symptoms from grade II. Outcomes were defined after 4 h since administration of antivenom or admission. Other monitored outcomes were death, complications of envenoming and adverse effects attributable to antivenom.
Study location	Tunisia
Belghith et al. 1999 [16]	
Methods	Retrospective analysis of a sub-population of patients enrolled for a clinical trial. Control group selected prospectively
Participants	Patients with scorpion stings older than 10 years. One hundred and thirty five patients who were administered bivalent antivenom for scorpion during a previous trial had their records re-examined and matched with controls on a pair-match basis.
Interventions	No prospective intervention. Controls did not receive antivenom.
Outcomes	Severity was graded as I (no systemic envenoming) and II (systemic envenoming). Prevention was defined as non-progression from grade I to II, and cure was defined as reversal of symptoms from grade II. Outcomes were defined after 4 h since administration of antivenom or admission. Other monitored outcomes were death, complications of envenoming and adverse effects attributable to antivenom.
Study location	Tunisia
Comparison	Antivenom plus prazosin vs. prazosin
Bawaskar et al. 2011 [23]	
Methods	Randomised open label clinical trial
Participants	Patients older than 6 months, reporting to hospital within 6 h of the sting and of grade II clinical severity (systemic autonomic symptoms without shock). A total of 70 patients were randomized, 35 each to test and control groups.
Interventions	The test group received Haffkine Biopharma monovalent scorpion antivenom (against *M. tamulus*) 30 ml dissolved in 100 ml of normal saline and infused over 30 min. Both groups received either 250 μg (under 18 years of age) or 500 μg of oral prazosin at 3 hourly intervals until the extremities were cold (resolution of peripheral vasodilatation).
Outcomes	Primary endpoint: resolution of grade II clinical syndrome at 10 h and prevention of progression to a higher grade (III, IV characterized by autonomic symptoms plus shock, pulmonary oedema) Secondary endpoints: time to total resolution of clinical syndrome, total dose of prazosin required within 10 h and adverse events
Study location	Maharashtra, India
Natu et al. 2010 [22]	
Methods	Prospective open label clinical trial
Participants	Patients aged 12–70 with a confirmed scorpion sting. The authors developed a composite clinical score based (minimum and maximum scores were 0 and 25, respectively) on pulse rate, blood pressure, presence of priapism, sweating, pain and neurological symptoms. Scores between 5 and 21 were included in the study. The enrolled were randomized into three trial arms: prazosin alone (*n* = 25), prazosin and antivenom (*n* = 28) and antivenom alone (*n* = 28).
Interventions	Haffkine monovalent antivenom was administered dissolved in distilled water (1:1) over 5–7 min intravenously. The total dose of antivenom was decided based on composite clinical score and patient’s age. The doses varied between 20 and 80 ml. Prazosin was administered orally at 3 hourly intervals at a dose of 500 μg (<20 kg body wt) or 1 mg (>20 kg body wt).
Outcomes	Primary endpoint: time to resolution of clinical symptoms
Study location	Maharashtra, India
Pandi et al. 2014 [24]	
Methods	Randomized controlled trial
Participants	Children aged less than 13 years were randomized into test (*n* = 25) and control (*n* = 25) groups. Grading of severity of sting was as according to Bawaskar et al.
Interventions	The test group received monovalent Haffkine antivenom according to protocol described by Bawaskar et al. Both groups received prazosin at a dose of 30 μg/kg/dose at 3 hourly intervals until resolution of the clinical syndrome.
Outcomes	Primary endpoint: time to resolution of clinical syndrome Secondary endpoints: total dose of prazosin required, adverse events, prevention of worsening of clinical syndrome and duration of hospital stay
Study location	Pondicherry, India

### Effects of interventions

#### Steroids vs. placebo

There is only a single trial that had addressed this comparison. Abroug et al. assessed the effect of steroids (intravenous hydrocortisone succinate 50 mg/kg, single dose or six hourly up to a total dose of 12 g per day) vs. placebo in Tunisia [3]. The commonly encountered venomous scorpion species in this region were *Androctonus australis* and *Buthus occitanus*. The trial was a well-designed prospective randomized controlled trial with a sample size of 600 patients (test arm—305, placebo arm—295). All patients were enrolled only after a confirmed scorpion sting (either seen or captured), and other medical treatment in both arms were similar except for the steroids. Scorpion antivenom had been administered for 25% of patients on the placebo arm and 20% of patients in the test arm. The severity of scorpion envenoming was graded as 1 (local envenoming) or 2 (systemic envenoming). Grade 2 envenoming was also comparable between the groups (16.5 vs. 17%). There was no significant difference in the overall hospital stay in either group (steroid group, 0.52 ± 0.6; placebo group, 0.58 ± 0.8 days). The occurrence of life-threatening complications of stings, namely, shock (two in each group) and pulmonary oedema (five in placebo group and seven in steroid group), were also similar among the groups. There were only two fatalities in the study, one in each study arm. Overall, it was concluded that administration of steroids did not have any added benefit. There are no other trials to re-confirm these findings independently.

### Pain relief

A single randomized controlled clinical trial had been carried out in Turkey to evaluate this important aspect of scorpion sting management [10]. They evaluated three methods of pain relief: paracetamol (1 g intravenous single dose), topical 5% lidocaine and ice application over the sting site. Pain in participants was assessed with a visual analogue scale (VAS) at 30th, 60th, 120th and 240th minutes. If pain was inadequately controlled after 30 min, intravenous pethidine was given irrespective of the randomization as rescue pain relief. A total of 130 patients were randomized to each of the three test arms, and none of them had systemic envenoming (six patients with systemic envenoming were excluded prior to randomization). There was no significant difference in the baseline VAS score in the three groups prior to pain relief. All three modalities reduced pain. However, topical lidocaine had a significant and superior analgesic effect compared to other two modalities at 30 min post application/administration (*p* < 0.001). There was no significant difference between the other two methods [10].

### Prazosin vs. supportive therapy

Evidence from observations and uncontrolled trials in the 1980s in India suggested a beneficial role of prazosin, a post synaptic alpha adrenergic blocker in mediating the ill effects of autonomic hyperactivity from scorpion stings [11, 12]. More specifically, these observations were on patients stung by the Indian red scorpion (*Mesobuthus tamulus*). It was suggested that prazosin would reduce the preload and afterload to counteract the hypertension caused by a catecholamine surge after a scorpion sting.

The only controlled trial that assessed the role of prazosin vs. no specific treatment is reported by Gupta in a paediatric cohort (mean age 4–5 years) in India [13]. It is a small study with 16 patients in the prazosin group and 20 in the control group. The test group received 30 μg/kg/dose on presentation and after 3 h. Further, doses were repeated 6 hourly in those with persistent evidence of systemic envenoming (e.g. pulmonary oedema). The author does not classify the enrolled subjects according to severity of envenoming, and the control group (treated with supportive care) is a historical cohort. Overall, the occurrence of complications (encephalopathy, pulmonary oedema, hyper/hypotension, myocarditis) did not show a significant difference between the two groups. However, the mortality (7 vs. 1) and mean duration of hospital stay (71.5 vs. 46.3 h) were significantly less in the prazosin-treated group.

A different study comparing intravenous dobutamine vs. prazosin (*n* = 21, in each treatment arm, all paediatric patients) concluded that recovery was faster (especially for complications such as pulmonary oedema) with prazosin [14].

### Antivenom vs. supportive therapy/placebo

We carried out two meta-analyses for this comparison with following inclusion criteria for study selection: (a) controlled studies evaluating antivenom vs. no antivenom for scorpion stings conducted up to 2015; (b) at least one arm of the study (the test arm) had to be prospectively enrolled (due to ethical reasons, it may be difficult to enrol a placebo arm prospectively); and (c) studies which had compared antivenom vs. prazosin were separately evaluated (see the ‘Antivenom vs. prazosin’ section). Following a previous meta-analysis by Abroug et al., old and new world scorpions were assessed separately [5]. The differences between this meta-analysis and ours are explained in the ‘Discussion’ section. The details of included studies are given in Table 2. The risk of bias is shown in Fig. 2.

#### Old world scorpions

In a trial published in 1999 from Tunisia, 825 consecutive patients were randomized into antivenom (*n* = 412) or placebo (*n* = 413) groups [15]. The test and control groups did not differ on demographic and clinical parameters at baseline. Clinical severity of sting was graded as I (absence of systemic manifestations) or II (presence of systemic manifestations). The medication they received prior to administration of antivenom/placebo was also similar and consisted mostly of steroids and antihistamines. Twenty millilitres of bivalent antivenom against *A. australis* and *B. occitanus* was administered for the test group over 15 min while the controls received a placebo. The investigators evaluated the participants at 4 h to decide on admission. The effect of treatment was evaluated as preventive (stalled the worsening from grade I to II) and cure (reversed manifestations from grade II to I). There was no difference in cure/prevention rates as well as admission rates at the end of 4 h of observation. For the admitted patients, subsequent duration of hospital stay was also similar between the groups. Two patients (one from each group) succumbed to envenoming.

Two other studies (not randomized prospective trials) with historical control groups have also suggested the futility of antivenom treatment in the late 1990s in Tunisia and Israel [16, 17]. Of these, the study by Sofer et al. (included in the meta-analysis by Abroug et al.) was excluded from further analysis as both ‘control’ and test groups were retrospective comparisons [17]. The study by Belghith et al. was included despite the antivenom group being a retrospective analysis but had been recruited as a part of a different clinical trial and hence expected to have well-maintained records [3, 16]. The control group for this study was prospectively recruited.

**Fig. 3:**
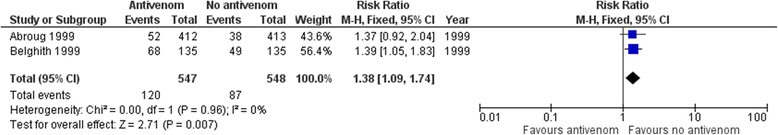
Forest plot for comparison of antivenom vs. placebo/supportive therapy for old world scorpion stings (outcome: symptom resolution at 4 h as an end point)

We conducted a meta-analysis of the studies by Belghith et al. and Abroug et al. using resolution of clinical syndrome after 4 h since administration of antivenom as an end point. There was no benefit of antivenom administration compared to supportive therapy. In fact, the pooled effect was significantly in favour of the control group (RR, 1.38; 95% CI, 1.09 to 1.74; 1095 participants; two trials) (Fig. 3).

Regarding adverse events, anaphylactic shock during antivenom administration was observed in a total of seven patients out of 547 patients (1.3%) (both trials combined). However, there were no deaths due to anaphylactic shock or other side effects attributable to antivenom.

#### New world scorpions

Three trials on new world scorpion stings met the inclusion criteria for inclusion in a meta-analysis [18–20]. Their methodological characteristics are discussed in Table 2, and risk of bias is shown in Fig. 2. A study included by Abroug et al. in their meta-analysis was excluded by us as both groups were retrospectively evaluated leading to a considerable degree of bias [21]. There was one new trial (which met the inclusion criteria) that had been published since the first meta-analysis [19].

The first of the included studies, conducted by Boyer et al. in Arizona, USA, showed a benefit of antivenom administration for *Centruroides* sp. stings [20]. This study was on children and had a very small sample size (*n* = 8 for antivenom group and *n* = 7 for placebo group). The antivenom used was developed against a venom mix of *Centruroides* sp. found in Mexico and was purported to be effective against *C. sculpturatus* prevalent in Arizona based on in vitro binding affinity studies. The outcomes assessed were resolution of clinical symptoms at 4 h after antivenom infusion (primary end point), and total dose of midazolam required to reduce agitation and plasma venom levels at 1 and 4 h (secondary end points). The group treated with antivenom showed a significant resolution of neurotoxicity at 4 h (*p* = 0.001) and a significantly lesser requirement for midazolam (measured as total cumulative dose administered at 4 h) (*p* = 0.01). Venom was undetectable in serum of all children treated with antivenom at 1 h while six children in placebo arm still had detectable venom (*p* = 0.001).

The second trial was also conducted by some of the authors in the previous trial, and they had expanded the scope of the trial to include centres in Mexico. The trial arm was recruited prospectively while the control arm was evaluated retrospectively probably due to ethical constraints. The study protocol for antivenom administration and outcomes assessed were similar to the previous study mentioned in the paragraph above. Seventy-eight patients received antivenom prospectively, and only two remained symptomatic at 4 h since administration. Of the historical cohort used as the control, a vast majority (93/97, 95.6%) still remained symptomatic at 4 h without antivenom. LoVecchio et al. carried out a prospective study without randomization for usefulness of anti-*Centruroides* sp. antivenom for stings in children less than 2 years [18]. In this study, 86 patients were administered antivenom while 46 were not. The mean recovery times of the two groups were 31 min (95% CI, 10–82 min) and 22.2 h (95% CI, 12–46 h), respectively [18].

There were no deaths reported in any of the three trials for new world scorpion stings in USA/Mexico. All subjects were children or young adults. LoVecchio et al. report serum sickness (defined as an unexplained rash within 1–21 days since administration of antivenom) in 49 (57%) patients. Other two trials do not mention any diagnoses of anaphylaxis or serum sickness. All adverse events recorded during follow-up are described as ‘mild’.

**Fig. 4:**
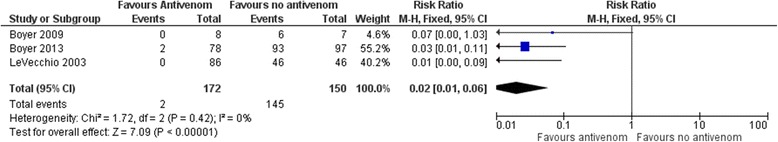
Forest plot for comparison of antivenom vs. placebo/supportive therapy for new world scorpion stings (outcome: symptom resolution at 4 h as an end point)

In our meta-analysis, for new world scorpions (*Centruroides* sp., using symptom resolution at 4 h as an end point), there was a clear benefit in favour of antivenom administration (RR, 0.02; 95% CI, 0.01 to 0.06; 322 participants; three trials) (Fig. 4).

### Antivenom vs. prazosin

The first published comparative study in this regard was by Bawaskar et al. in 2007, comparing antivenom (*n* = 25) against prazosin (*n* = 28). This was not a randomized study. The antivenom had been administered in peripheral healthcare centres prior to arrival at study hospital (which had no involvement with the study), and variable doses of antivenom had been given to patients without a proper trial protocol. The patients arriving directly at the study centre were treated with prazosin (without antivenom). There were four deaths in the antivenom-treated group vs. no deaths in the prazosin-treated group. The mean recovery time was also longer in the antivenom-treated group. Authors state that the mean time lapse from sting to arrival at hospital and degree of envenoming (measured by number of patients with autonomic disturbances at presentation) were reasonably similar between the two groups. However, overall, this comparison provides poor quality evidence due to methodological faults.

In contrast, in the prospective controlled trial by Natu et al. (described below and in Table 2), there were two trial arms treated with prazosin alone and antivenom alone (in addition to a third trial arm treated by a combination of antivenom and prazosin) [22]. In comparison, the antivenom-treated group had a significantly improved recovery time compared to the prazosin-treated group (mean difference, −15.1 h; 95% CI, −17.2 to −13.1). Regarding quality of evidence as per study design, this observation is much stronger than the previous observation. It is also keeping in line with the overall trend shown on the next comparison.

### Antivenom plus prazosin vs. prazosin

For this comparison, all the available evidence is for old world scorpion stings and from India. The commonest venomous scorpion species associated with these trials is Indian red scorpion (*M. tamulus*).

There were three trials that had compared antivenom and prazosin against prazosin alone. They had also measured at least one comparable outcome (mean duration to resolution of clinical symptoms). All three were prospective controlled studies within the same geographical region treating the same type of scorpion sting which reduced heterogeneity. The details of the studies are given in Table 2. The common risk of bias figure demonstrates the risk of bias of each study.

Two trials had enrolled both adults and children [22, 23] while one trial was restricted to children [24]. All trials had used the same antivenom against *M. tamulus* from the same manufacturer, and the dosage and administration protocol was identical in two trials [23, 24]. These two trials (Bawaskar et al. and Pandi et al.) have also categorized severity grading of the envenoming using a similar scale [23, 24]. The other trial by Natu et al. have made their own clinical composite score to assess the severity of envenoming, but that is also based on the same autonomic disturbances, and they have excluded individuals with local envenoming only and severe envenoming. Therefore, the included patients are approximately comparable across the studies with regard to clinical severity. The antivenom doses used by Natu et al. had a wider range than the fixed dose used by other two trials.

**Fig. 5:**
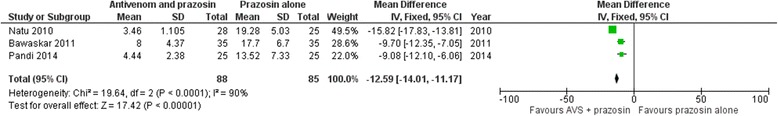
Forest plot for comparison of antivenom and prazosin vs. prazosin alone for *M. tamulus* stings (old world scorpions, outcome: mean duration for symptom resolution)

A meta-analysis of all three trials (common outcome of mean duration to resolution of the clinical syndrome) confirmed the superiority of combined therapy vs. prazosin alone (mean difference, −12.59 h; 95% CI, −14.01 to −11.17; 173 participants; three trials) (Fig. 5).Of the other outcomes reported, Bawaskar observed a significant reduction in the total number of prazosin doses required when the drug was combined with antivenom (mean difference, −2.0; 95% CI, −2.5 to −1.6) [23]. There was no significant difference in prevention of worsening to a higher grade of envenoming between the two groups though the numbers were more in the prazosin only group. Pandi et al. also observed significant improvements for both these outcomes in the combination treatment group [24]. Natu et al. do not report on these outcomes. There were no deaths in any of the trials and no significant adverse events to antivenom administration. Natu et al. have observed precipitation of pulmonary oedema in three patients with baseline hypertension when treated with prazosin.

## Discussion

### Steroids vs. placebo

The futility of steroids in scorpion envenoming is probably explained from the fact that toxins do not induce an extensive immune reaction in the immediate aftermath (within 4 h when a majority manifest systemic envenoming). The envenoming is a result of functional disturbance of voltage-gated sodium ion channels that lead to autonomic hyper-excitability. Adrenal insufficiency, another mechanism via which steroids were purported to show benefit, has not been confirmed in humans following scorpion stings [3]. Overall, given the similarity of study arms of the quoted trial with respect to confounding factors as well as the large sample size, the evidence from this study can be considered as good quality evidence.

### Pain relief

The study by Aksel et al. was a well-designed prospective randomized trial with an adequate sample size. However, it has two inherent sources of bias due to non-blinding (which is probably unavoidable) and exclusion of patients with severe envenoming. Doctors and probably patients also knew that lidocaine is a potent local anaesthetic agent and that might have biased the VAS scoring in favour of lidocaine. Also, exclusion of severe envenoming limits the interpretation of results to mild envenoming only. The pain perception and hence response to analgesia in severe envenoming can vary significantly from mild envenoming. Finally, the use of VAS to report pain is highly subjective as different people perceive pain differently. However, randomization would have helped (at least partially) to offset this bias.

### Prazosin vs. supportive therapy

The quality of evidence of the study by Gupta et al. [13] is not strong from a methodological point of view given the small sample size and the lack of a randomized control group that was simultaneously enrolled. However, from an ethical point of view, it would have been difficult to design a study that would not offer any treatment against prazosin (given the prior observations of its purported benefit). The status quo remains to this date for this comparison. However, as mentioned in the sections below, there is indirect evidence for a beneficial role of prazosin in *M. tamulus* stings especially when it is used as add-on therapy to antivenom.

### Antivenom vs. supportive therapy/placebo

Given the heterogeneity of study designs (historical analysis vs. prospective controlled trials) and the taxonomical differences in scorpion species concerned, it is difficult to combine studies in a meta-analysis. One alternative is to follow the approach adopted by an earlier meta-analysis published in 2011 by Abroug et al. which makes a clear distinction about antivenom against old world and new world scorpions [5]. In this meta-analysis (Abroug et al.), there was a significant improvement of symptoms with antivenom against new world *Centruroides* sp. (risk difference, 0.53; 95% CI, 0.16 to 0.91; *p* < 0.001), but there was no such effect against old world scorpions (risk difference, −0.05; 95% CI, −0.28 to 0.18; *p* = 0.65). The studies included in old world scorpions had been conducted in Tunisia, Israel and India [15–17, 22, 25]. However, there were some methodological concerns regarding the included studies which could have affected the quality of evidence. For example, it had included studies where both test and control arms were historical comparisons based on records. It was also noted that some studies included in the first meta-analysis had in fact compared antivenom against prazosin, a treatment that probably has merit on its own [22, 25]. Multiple sources of bias (selection, detection, reporting bias) and non-uniform data for outcome reporting reduced the quality of evidence from this meta-analysis which we attempted to avoid in our analysis (e.g. trials that had prazosin as a treatment are evaluated separately). The inclusion criterion for our meta-analysis was described above.

Despite the more refined meta-analysis, the results observed by us and Abroug et al. for this comparison were similar. Our meta-analysis also confirmed a significant benefit of symptom resolution with antivenom against *Centruroides* sp. stings (new world scorpions) but not with antivenom used against old world scorpions in Tunisia. While trials for new world scorpions were recent, those for old world scorpions were conducted more than 15 years ago and from a single country. There is plenty of scope to improve on this by further trials covering different geographical locations and different scorpion species. This should be a priority in toxinological research.

### Antivenom vs. prazosin

There are two studies that had assessed this comparison with conflicting observations. Findings by Bawaskar et al. favour prazosin while Natu et al. favour antivenom. However, due to the methodological shortcomings in the study by Bawaskar et al., the observations of Natu et al. are more acceptable.

### Antivenom plus prazosin vs. prazosin

There were three trials for this comparison all concerning *M. tumulus* stings in India. They had used the same antivenom preparation and assessed similar outcome measures that were comparable to combine in a meta-analysis which showed a significant benefit for the antivenom and prazosin combination. All the trials were prospective controlled trials, and two of them were randomized trials [23, 24] (randomization process is not mentioned in the trial by Natu et al.). Age and sex of participants as well as their baseline autonomic disturbances were reported in all the trials and were not found to be different between the groups. Therefore, this evidence can be considered as of good quality for this therapeutic comparison against *M. tamulus* stings in India.

### Limitations

The methodological quality of most trials on scorpion sting management does not meet the gold standard of double blind randomized controlled studies. However, ethical obligations and challenges in designing a blinded randomized trial have to be taken into account with regard to scorpion stings. The interpretations and recommendations regarding scorpion sting management are restricted to the specific scorpion species (and hence their geographical location) studied. From the available data, useful recommendations can only be drawn for *Centruroides* species in USA and Mexico and *M. tamulus* in India.

## Conclusions

Following conclusions can be summarized from all the evidence presented above on various aspects of management of scorpion stings for both old and new world scorpion stings. The ‘antivenom’ mentioned below is not a uniform entity. The trials have been carried out in only a few countries, and recommendations are hence valid within the context of these locations, scorpion species and age group of participants. Therefore, we have indicated these parameters in addition to the level of evidence [26] within brackets of each recommendation.

For old world scorpions,

- Steroids have no benefit in management of scorpion stings (level of evidence, 2b; location, Tunisia; common species, *A. australis, B. occitanus;* applicable age group, children and adults over 10 years).
- Local anaesthesia by topical lidocaine patches may be superior to intravenous paracetamol or local ice application for pain relief in mild envenoming (level of evidence, 1b; location, Turkey; common species, *Androctonus crassicauda, Leiurus quinquestriatus, Mesobuthus gibbosus and Mesobuthus eupeus*; applicable age group, adults over 18 years).
- Prazosin may be better than supportive therapy alone for stings by *M. tamulus* (level of evidence, 3b; location, India; applicable age group, in children).
- Polyvalent antivenom against *A. australis* and *B. occitanus* were ineffective when compared to placebo (level of evidence, 2a; location, Tunisia; applicable age group, children and adults over 10 years).
- Antivenom against *M. tamulus* may be better than prazosin alone (level of evidence, 2b; location, India; applicable age group, over 12 years of age).
- Antivenom (against *M. tumulus)* and prazosin combination is better than prazosin alone (level of evidence, 1b; location, India; applicable age group, both adults and children over 6 months of age).

For new world scorpions,

- Antivenom against *Centruroides* sp. are effective in reversing the clinical syndrome faster than no antivenom (level of evidence, 2a; location, Mexico and USA; applicable age group, children and young adults aged less than 18 years).

## Additional file

Additional file 1: The PRISMA checklist. The PRISMA checklist for this review. (DOCX 28 kb)
